# Cost-effectiveness of additional serplulimab to chemotherapy in metastatic squamous non-small cell lung cancer patients

**DOI:** 10.3389/fimmu.2024.1382088

**Published:** 2024-04-22

**Authors:** Hanrui Zheng, Ya Zeng, Feng Wen, Ming Hu

**Affiliations:** ^1^ West China School of Pharmacy, Sichuan University, Chengdu, China; ^2^ Department of Clinical Pharmacy, West China Hospital, Sichuan University, Chengdu, China; ^3^ Department of Medical Oncology, Cancer Center, West China Hospital, Sichuan University, Chengdu, China; ^4^ West China Biomedical Big Data Center, Sichuan University, Chengdu, China

**Keywords:** cost-effectiveness, serplulimab, chemotherapy, squamous non-small cell lung cancer, partitioned survival model

## Abstract

**Objective:**

To estimate the cost-effectiveness of adding serplulimab to chemotherapy for metastatic squamous non-small cell lung cancer (NSCLC) patients in a first-line setting from a Chinese perspective.

**Methods:**

A three-health state partitioned survival model was constructed to simulate disease development. The clinical data used in the model were derived from the ASTRUM-004 clinical trial. Only direct medical costs were included, and the utilities were derived from published literature. The quality-adjusted life-years (QALYs) and incremental cost-effectiveness ratio (ICER) were employed to evaluate health outcomes. Additionally, a sensitivity analysis was performed to verify the robustness of the results.

**Results:**

Compared with chemotherapy alone, the addition of serplulimab resulted in an increase of 0.63 QALYs with an incremental cost of $5,372.73, leading to an ICER of $8,528.14 per QALY. This ICER was significantly lower than 3 times China’s per capita GDP. The one-way sensitivity analysis suggested that the utility of PFS was the most sensitive factor on ICERs, followed by the price of serplulimab.

**Conclusion:**

The combination of serplulimab and chemotherapy has been shown to be a cost-effective initial treatment option for patients with metastatic squamous NSCLC with the commonly accepted willingness-to-pay threshold of 3 times the GDP per capita per QALY in China.

## Introduction

Lung cancer remains the leading cause of cancer-related mortality worldwide (1). The associated economic burden makes it a serious public health concern, particularly in Asia, where it carries the heaviest economic burden compared to other cancer types (2, 3). Non-small cell lung cancer (NSCLC) is the most common subtype of lung cancer and accounts for approximately 80% of lung cancer cases (4). The survival of metastatic NSCLC patients has significantly improved due to the rapid progression of targeted drugs (5).

Nevertheless, EGFR mutations or ALK rearrangements are uncommon in squamous NSCLC, which accounts for approximately 25%-30% of all NSCLC cases, and is linked with a worse prognosis in comparison to lung adenocarcinoma (6). Immunotherapy presents a potential alternative to traditional treatment methods for patients lacking gene mutations or alterations. The substantial advancements achieved with immune checkpoint inhibitors have notably revolutionized the medical management of lung cancer, fostering a more effective and personalized approach (7). Pembrolizumab was the first programmed cell death protein-1(PD-1) inhibitor approved by the Food and Drug Administration (FDA) combined with chemotherapy for metastatic squamous NSCLC as a first-line treatment (8). Several domestic PD-1 inhibitors, such as sintilimab, tislelizumab, and camrelizumab, have received approval from the National Medical Products Administration (NMPA) for use in combination with chemotherapy for treating metastatic squamous NSCLC (9–11). However, the approval of these inhibitors was based on clinical studies that exclusively involved Chinese patients with metastatic squamous NSCLC. The Phase III IMpower131 trial reported no significant improvement in the overall survival (OS) of patients with metastatic squamous NSCLC when atezolizumab was added to platinum-based chemotherapy (12). Hence, there continues to be a global shortage of immune checkpoint inhibitors for patients with advanced squamous NSCLC.

In a recent study, the randomized, double-blind, phase III trial ASTRUM-004, conducted at 85 hospitals and academic research centers across 6 countries, assessed the efficacy of combining serplulimab with platinum-based chemotherapy in previously untreated patients with metastatic squamous NSCLC (13). Serplulimab significantly prolonged the median progression-free survival (PFS) by 2.6 months (8.3 months vs 5.7 months, hazard ratio (HR), 0.53; 95% confidence interval (CI), 0.42–0.67), and the median OS by 4.5 months (22.7 months vs. 18.2 months, HR, 0.73, IC, 0.58–0.93). Thus, serplulimab was approved in combination with chemotherapy for patients with metastatic squamous NSCLC by the NMPA in China.

Despite the considerable efficacy of serplulimab, its cost and occurrence of immune-related adverse events (AEs) may impose an additional financial burden on patients with metastatic squamous NSCLC. Hence, it has become essential to assess the economic implications of serplulimab. The aim of our analysis was to evaluate the cost-effectiveness of combining serplulimab with chemotherapy for metastatic squamous NSCLC patients without EGFR or ALK genomic tumor aberrations from the perspective of the Chinese healthcare payer.

## Methods

### Clinical information

The criteria for inclusion in the ASTRUM-004 trial included patients diagnosed with stage IIIB-IV squamous NSCLC, aged 18 or older, lacking known EGFR mutations or ALK/ROS1 fusions, and without a history of systemic therapy for metastatic disease (13). In total, 809 patients were enrolled and randomized at a 2:1 ratio into two treatment groups. The treatment regimens for metastatic squamous NSCLC patients included the administration of carboplatin (at an area under the curve of 5-6 mg/mL per min on day 1) and albumin-bound paclitaxel (100 mg/m^2^ on days 1, 8, and 15) during a 3-week cycle for 4-6 cycles. Additionally, patients received either serplulimab (4.5 mg/kg on day 1) or a placebo for a maximum period of 2 years. The median follow-up duration was 31.1 months (range 0.2–41.6). Patients in the trial group received a median of 9.0 cycles of serplulimab. In the serplulimab plus chemotherapy group, patients received a median of 11.0 cycles of nab-paclitaxel, while in the placebo plus chemotherapy group, the median number of cycles was 10.0. Additionally, both groups of patients received a median of 4 cycles of carboplatin treatment.

### Model overview and model transition probabilities

**Figure 1:**
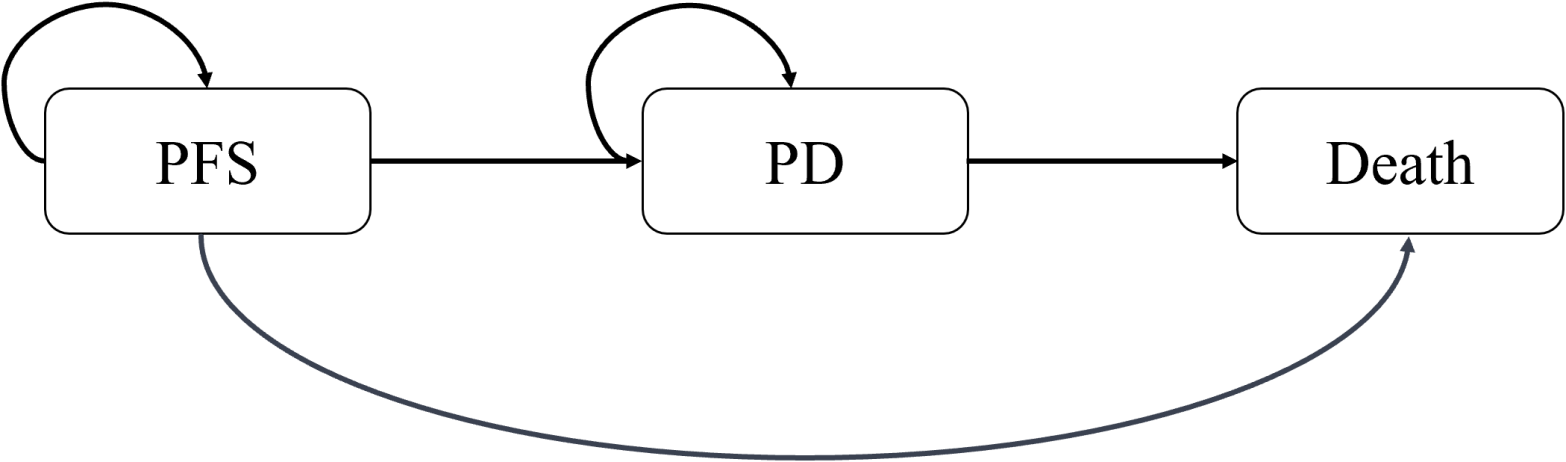
The structure of the partitioned survival model for metastatic squamous NSCLC. PFS, progression-free. PD, progression.

A partitioned survival model was constructed using TreeAge Pro 2020 software (TreeAge, Williamstown, MA, USA) to estimate the cost-effectiveness of adding serplulimab to chemotherapy regimen. Three exclusive states were included in the model: PFS, progressed disease (PD), and death (**Figure 1**). One month was set as a cycle in the model and the time horizon was 10 years. Quality-adjusted life years (QALYs) and incremental cost-effectiveness ratios (ICERs) were calculated as health outcomes.

**Figure 2:**
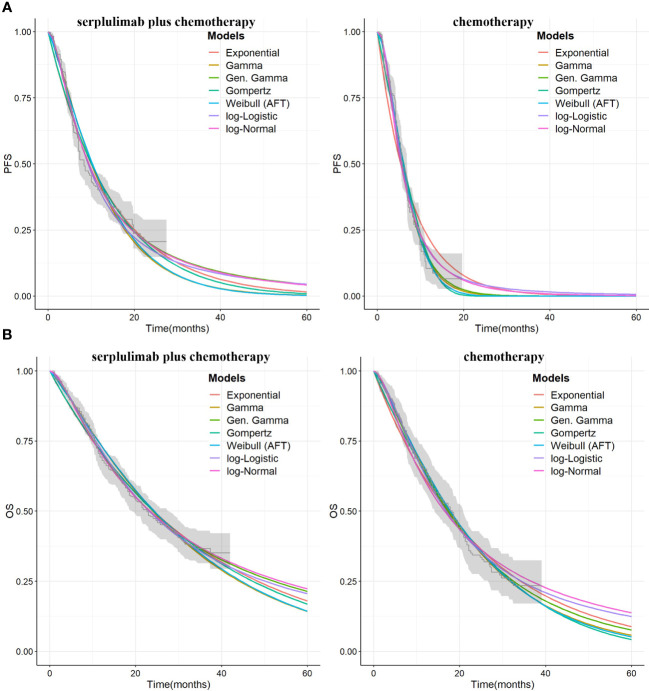
**(A)**The exploration of survival curves for PFS. **(B)** The exploration of survival curves for OS. PFS, progression-free. OS, overall survival.

The Kaplan-Meier survival information for PFS and OS was derived from the ASTRUM-004 trial using the GetData graph digitizer (version 2.26, http://getdata-graph-digitizer.com/). Subsequently, individual patient data were reconstructed using the digitized R package (14). Survival probabilities were estimated using various distribution models, including Weibull, log-logistic, log-normal, gompertz, exponential, gengamma and gamma models. The optimal fitting distribution was evaluated through the Akaike information criterion (AIC) and the Bayesian information criterion (BIC). The results of the survival curve simulation are shown in **Figure 2** and **Table 1**. The best fitting distribution parameters for the PFS and OS data in the combination group were lognormal distributions. The most appropriate distribution parameters for the PFS and OS data in the chemotherapy alone group were the gamma and log-logistic distributions, respectively.

**Table 1:** Results of the statistical fit to the survival curves in the ASTRUM-004 trial.

	PFS of serplulimab plus chemotherapy		PFS of chemotherapy		OS of serplulimab plus chemotherapy		OS of chemotherapy	
	AIC	BIC	AIC	BIC	AIC	BIC	AIC	BIC
**Exponential**	1479.701	1483.582	712.7836	715.9710	1778.574	1782.455	970.3699	973.5573
**Gamma**	1465.691	1473.452	680.7112	687.0860	1774.372	1782.133	966.9884	973.3632
**Gengamma**	1450.817	1462.458	682.6012	692.1633	1768.192	1779.833	968.5855	978.1477
**Gompertz**	1481.533	1489.294	696.4411	702.8159	1780.513	1788.274	970.7714	977.1462
**Weibull**	1471.203	1478.964	682.5996	688.9740	1776.264	1784.025	967.6322	974.0070
**Loglogistic**	1449.581	1457.342	684.5288	690.9036	1767.397	1775.158	966.9508	973.3256
**Lognormal**	1448.846	1456.607	684.9435	691.3183	1766.373	1774.134	972.4324	978.8072

PFS, progression-free survival; OS, overall survival; AIC, Akaike information criterion; BIC, Bayesian information criterion.

### Cost and utility estimates

Direct costs, including the cost of medicine, cost of supportive care, cost of terminal care, and management cost of necessary AEs, were calculated from the Chinese payer perspective. In our model, we assumed that the average weight of the patients was 65 kg, with an average body surface area of 1.8m^2^ and a creatinine clearance rate (CCR) of 90 ml/min/1.73 m^2^ (15). The price of serplulimab and the prices of chemotherapy were sourced from the Chinese Drug Bidding Database (https://www.yaozh.com/). The costs associated with supportive care, drug administration, and terminal care were obtained from published literature (16–18). Metastatic squamous NSCLC patients in the placebo group were eligible to receive serplulimab monotherapy treatment after disease progression. Due to the limited information on second-line therapies, we assumed that the other patients had been treated with nivolumab, tislelizumab, or docetaxel according to clinical guidelines (19). All costs were converted to US dollars ($1 = ￥7.21), and the details of costs are presented in **Table 2**. The health utility values used in our study were all from the published literature. The utility value in the PFS state was 0.86, and the utility in the PD state was 0.32 (20, 21). Additionally, we determined the disutility values associated with AEs from previous literature (20, 22). The annual discount rate for both costs and utilities was set at 5%. As per the World Health Organization (WHO) recommendation, the willingness-to-pay (WTP) threshold was set at three times the gross domestic product (GDP) per capita in China ($38,052/QALY).

**Table 2:** Major parameters in the model and the ranges of parameters.

Parameters	Value	Distribution	Source
Costs ($)			
** Cost of drugs per 1mg**			
Serplulimab	0.77	Gamma	Local database
Carboplatin	0.15	Gamma	Local database
Albumin-bound paclitaxel	0.52	Gamma	Local database
**Supportive care per cycle** ^§^	72	Gamma	(16)
**Cost of drug** **administration** **per unit**	19.11	Gamma	(23)
** Cost of AEs per cycle**			
Serplulimab plus chemotherapy	36.60	Gamma	Local database
Chemotherapy	97.31	Gamma	Local database
**Cost of terminal care** **per patient** ^*^	16,441.83	Gamma	(18)
** Subsequent treatment cost per cycle**			
Serplulimab plus chemotherapy	55.94	Gamma	Local database
Chemotherapy	76.06	Gamma	Local database
Utilities			
PFS	0.86	Beta	(20, 21)
PD	0.32	Beta	(20, 21)
**Disutility of AE**		Beta	
Anemia	0.07	Beta	(22)
White blood cell count decreased	0.20	Beta	(20)
Neutrophil count decreased	0.20	Beta	(20)
Platelet count decreased	0.11	Beta	(24)
Survival models for serplulimab plus chemotherapy			
Lognormal model for PFS	Log-mean = 2.2457, log-SD = 1.0671		(13)
Lognormal model for OS	Log-mean =3.1493, log-SD = 1.2373		(13)
Survival models for chemotherapy			
Gamma model for PFS	Shape = 2.0773, rate = 0.3013		(13)
Log-logistic model for OS	Shape = 1.5350, rate = 16.7842		(13)

PFS, progression-free survival; PD, progressive disease; AEs, adverse events;OS, overall survival.

§Physician visits, laboratory tests, and examinations were all included in the routine follow-up cost

*Overall total cost per patient regardless of treatment duration.

### Sensitivity analysis

The robustness of the model was verified using one-way sensitivity analysis and probabilistic sensitivity analysis. In the one-way sensitivity analysis, model parameters were adjusted within a range of ±20% from their baseline values. For the probabilistic sensitivity analysis, 1,000 Monte Carlo simulations were conducted using parameters with specific probability distributions. A cost-effectiveness acceptability curve was generated to illustrate the ICERs across different WTP thresholds.

## Results

### Base case results

As shown in **Figure 1**, a partition survival model was built to assess the cost effectiveness of serplulimab plus chemotherapy and chemotherapy alone. The base case results of the two treatment regimens are presented in **Table 3**. Compared with chemotherapy alone, the addition of serplulimab resulted in an additional 0.63 QALYs (1.65 versus 1.02 QALYs), with costs increased by $5,372.73 ($10,155.45 versus $4,782.72), leading to an ICER of 8,528.14 per QALY. The ICER was lower than the WTP threshold, suggesting that the use of serplulimab for the first-line treatment of metastatic squamous NSCLC was cost-effective.

**Table 3:** Results in the base-case analysis.

Result	Serplulimab plus chemotherapy group		Chemotherapy group
**Cost ($)**	10,155.45		4,782.72
**Incremental costs**	5,372.73		
**QALYs**	1.65		1.02
**Incremental QALYs**	0.63		
**ICER ($/QALY)**	8,528.14		

QALY, quality-adjusted life year; ICER, Incremental cost-effectiveness ratio.

### Sensitivity analysis

**Figure 3:**
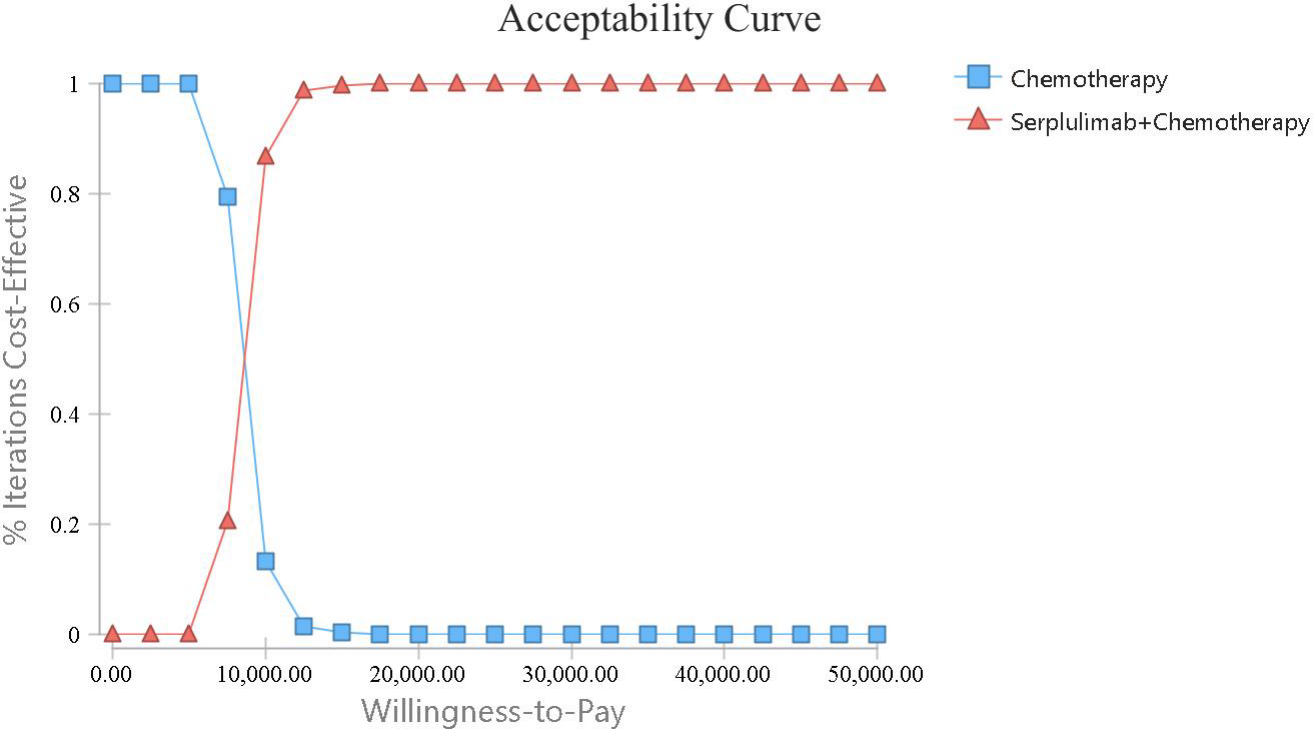
The cost-effectiveness acceptability curve comparing the addition of serplulimab to chemotherapy with chemotherapy alone.

**Figure 4:**
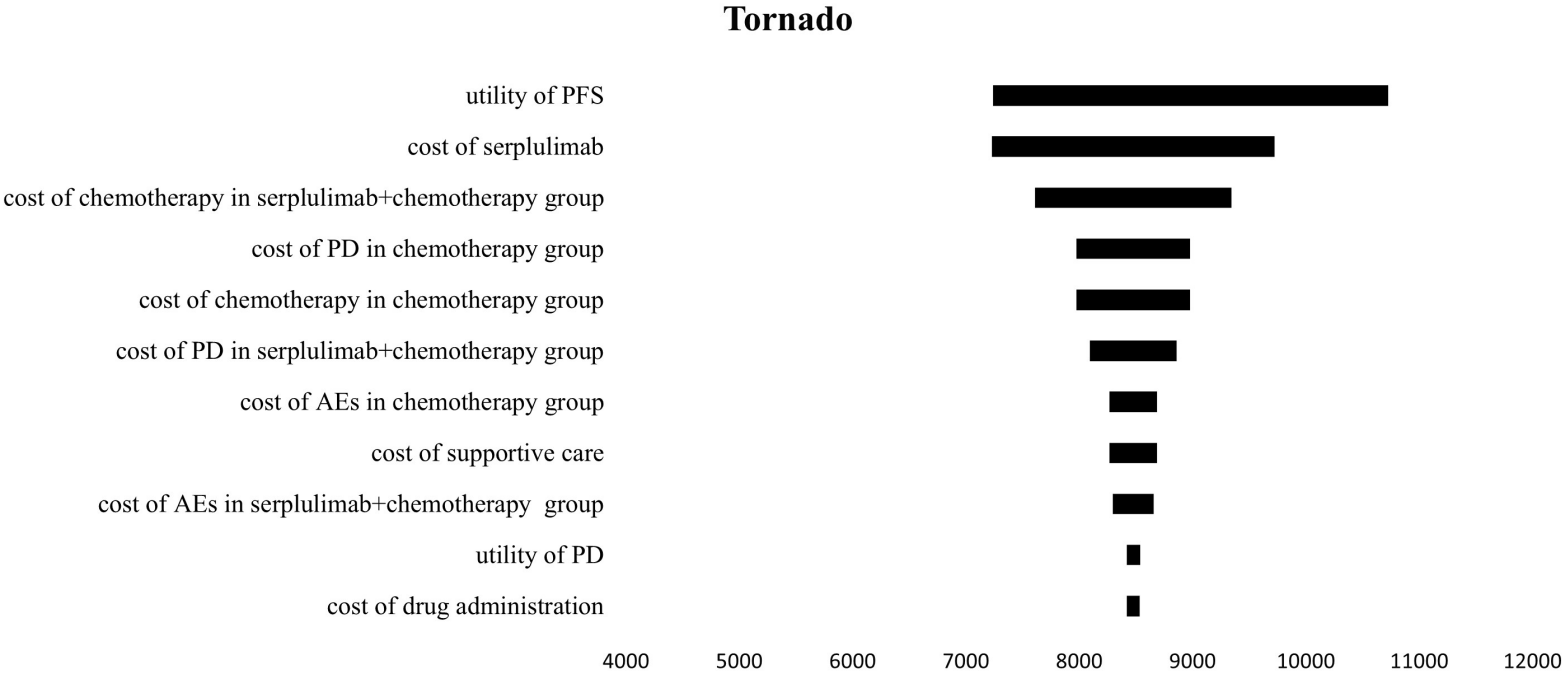
The Tornado diagram.PFS, progression-free survival; PD, progressive disease; AEs, adverse events.

A one-way sensitivity analysis was conducted to explore the effects of parameter variations on the analysis results in this model. The findings are visualized in a tornado diagram (**Figure 3**), revealing that the utility of PFS and the cost of serplulimab had the most substantial impact on the ICER. Conversely, the costs associated with treating AEs and the expenses related to supportive care in both treatment regimens had a lesser impact on the ICER. Overall, the ICER of serpalimab plus chemotherapy compared with chemotherapy alone was less than $38,052 per QALY, despite fluctuations in individual parameters. The cost-effectiveness acceptability curve is shown in **Figure 4**, which illustrates the cost-effectiveness probability of serplulimab plus chemotherapy under different WTP thresholds. The cost-effectiveness acceptability curve indicated that at a threshold of $17,500, the probability of serplulimab plus chemotherapy being cost-effective was 99.9%.

## Discussion

Lung cancer contributes to approximately 25% of all cancer disability-adjusted life years (DALYs), with the greatest DALY burden in China (25). NSCLC, which includes squamous and nonsquamous cell carcinomas, is the most prevalent type of lung cancer, and each of these types of NSCLC has distinct biological characteristics (4). Squamous NSCLC is generally more aggressive than nonsquamous NSCLC (26). The difference in the immune microenvironment between squamous and nonsquamous NSCLC may impact immunotherapy outcomes in patients treated with PD-1/PD-L1 inhibitors (27). On a global scale, there is still a relative shortage of immunotherapeutic drugs available for treating metastatic squamous NSCLC. The ASTRUM-004 trial, an international multicenter phase III study, demonstrated the efficacy of serplulimab in metastatic squamous NSCLC, thereby presenting a novel treatment option for these patients. There is an urgent need to assess the cost-effectiveness of serplulimab in metastatic squamous NSCLC.

To the best of our knowledge, our study represents the first cost-effectiveness analysis of combining serplulimab with chemotherapy for metastatic squamous NSCLC patients in China. Our analysis indicated that the addition of serplulimab to chemotherapy was cost-effective, with an ICER of $8,528.14 per QALY, which was significantly lower than the WTP threshold. The model’s robustness was confirmed through one-way sensitivity and probabilistic sensitivity analyses. When the threshold of WTP was set as one time the GDP per capita, the probability of cost-effectiveness of serplulimab plus chemotherapy exceeded 99.0%. The one-way sensitivity analysis demonstrated that the utility of PFS was the most sensitive factor, followed by the cost of serplulimab. When utility of PFS ranged from 0.688 to 1, the ICERs ranged from $7,242.32 to $10,725.64, which was also below the WTP. Currently, serplulimab is not covered by health insurance in China, but there is a charitable drug donation program. If serplulimab is included in medical insurance through price negotiations, its price is expected to decrease in the future. Overall, serplulimab presents an affordable treatment option for Chinese metastatic squamous NSCLC patients.

The financial burden associated with treating lung cancer is substantial, often exceeding the annual household incomes of several families (28). In recent years, the emergence of immunotherapy drugs has significantly transformed cancer treatment. However, the use of these innovative drugs raises costs, further compounding the economic burden on families and society (29). To reduce the cost of cancer treatment, China has implemented a series of policies, including price negotiations. Following national price negotiations, the prices of innovative anti-cancer drugs have significantly decreased, thereby enhancing accessibility and affordability (30). By 2021, camrelizumab, sintilimab, and tislelizumab were included in the National Medical Insurance List in China (31). A decrease in the price of domestic PD-1 inhibitors plays a crucial role in diminishing national healthcare spending and easing the financial burden on patients. The addition of camrelizumab to chemotherapy was considered cost-effective from a healthcare perspective in China for metastatic NSCLC patients (32). Zhao et al. discovered that the combination of camrelizumab with paclitaxel-based chemotherapy, followed by docetaxel, within a selection of 11 treatment sequences for metastatic NSCLC patients, represented a cost-effective treatment option and tislelizumab was deemed the most effective and cost-effective second-line option (33). The combination of sintilimab and chemotherapy was proven to be more cost-effective than the combination of pembrolizumab and chemotherapy for patients with metastatic NSCLC in China, as the QALYs obtained in the sintilimab group were similar to those in the pembrolizumab group, with substantially lower costs (34).

Our research has the following limitations. First, as the utilities of patients treated with serplulimab have not been publicly reported, we obtained the utilities in our study from previous literature. The sensitivity analysis revealed that the PFS utility was the main influencing factor on the results, so it may have a certain impact on the model results. However, fluctuations in PFS within a range of ±20% did not affect the results of the study. Second, due to the lack of data on subsequent treatment in the ASTRUM-004 trial, we assumed that patients in this model received second-line treatment recommended by guidelines, which may not reflect clinical practice. Third, the use of parametric simulations to extrapolate PFS and OS for long-term survival introduced uncertainty. Nevertheless, the best fit parameter model was selected based on the AIC and BIC criteria. Forth, although sensitivity analyses demonstrated the robustness of the results, the differences in costs and health care in different countries may affect the validity and generalizability of our results. Finally, our analysis primarily depended on data from the ASTRUM-004 trial. In the real world, clinical outcomes may be influenced by a variety of factors, including organ function, medication adherence, and race. The effectiveness and economy of serplulimab in advanced squamous NSCLC patients still needs long-term follow-up and validation in clinical practice. Despite these limitations, our results have great significant of the selection of medicine for metastatic squamous NSCLC in China.

## Conclusion

In conclusion, the addition of serplulimab to chemotherapy, compared with chemotherapy alone, is cost-effective for first-line treatment of metastatic squamous NSCLC from the perspective of the Chinese health system. The study’s conclusions are beneficial for guiding clinical decision-making and allocating health resources.

## Data availability statement

The original contributions presented in the study are included in the article/supplementary material. Further inquiries can be directed to the corresponding author.

## Author contributions

HZ: Conceptualization, Data curation, Formal analysis, Software, Writing – original draft. YZ: Data curation, Visualization, Writing – review & editing. FW: Formal analysis, Project administration, Writing – review & editing. MH: Investigation, Supervision, Validation, Writing – review & editing.
